# Identification of key monocytes/macrophages related gene set of the early-stage abdominal aortic aneurysm by integrated bioinformatics analysis and experimental validation

**DOI:** 10.3389/fcvm.2022.950961

**Published:** 2022-09-14

**Authors:** Shuai Cheng, Yuanlin Liu, Yuchen Jing, Bo Jiang, Ding Wang, Xiangyu Chu, Longyuan Jia, Shijie Xin

**Affiliations:** ^1^Department of Vascular and Thyroid Surgery, The First Hospital, China Medical University, Shenyang, China; ^2^Key Laboratory of Pathogenesis, Prevention, and Therapeutics of Aortic Aneurysm in Liaoning Province, Shenyang, China; ^3^Regenerative Medicine Research Center of China Medical University, Shenyang, China

**Keywords:** bioinformatics, abdominal aortic aneurysm, macrophage, single-cell RNA sequencing, WGCNA

## Abstract

**Objective:**

Abdominal aortic aneurysm (AAA) is a lethal peripheral vascular disease. Inflammatory immune cell infiltration is a central part of the pathogenesis of AAA. It’s critical to investigate the molecular mechanisms underlying immune infiltration in early-stage AAA and look for a viable AAA marker.

**Methods:**

In this study, we download several mRNA expression datasets and scRNA-seq datasets of the early-stage AAA models from the NCBI-GEO database. mMCP-counter and *CIBERSORT* were used to assess immune infiltration in early-stage experimental AAA. The scRNA-seq datasets were then utilized to analyze AAA-related gene modules of monocytes/macrophages infiltrated into the early-stage AAA by Weighted Correlation Network analysis (WGCNA). After that, Gene Ontology (GO) and Kyoto Encyclopedia of Genes and Genomes (KEGG) functional enrichment analysis for the module genes was performed by ClusterProfiler. The STRING database was used to create the protein-protein interaction (PPI) network. The Differentially Expressed Genes (DEGs) of the monocytes/macrophages were explored by Limma-Voom and the key gene set were identified. Then We further examined the expression of key genes in the human AAA dataset and built a logistic diagnostic model for distinguishing AAA patients and healthy people. Finally, real-time quantitative polymerase chain reaction (RT-qPCR) and Enzyme Linked Immunosorbent Assay (ELISA) were performed to validate the gene expression and serum protein level between the AAA and healthy donor samples in our cohort.

**Results:**

Monocytes/macrophages were identified as the major immune cells infiltrating the early-stage experimental AAA. After pseudocell construction of monocytes/macrophages from scRNA-seq datasets and WGCNA analysis, four gene modules from two datasets were identified positively related to AAA, mainly enriched in Myeloid Leukocyte Migration, Collagen-Containing Extracellular matrix, and PI3K-Akt signaling pathway by functional enrichment analysis. *Thbs1*, *Clec4e*, and *Il1b* were identified as key genes among the hub genes in the modules, and the high expression of *Clec4e*, *Il1b*, and *Thbs1* was confirmed in the other datasets. Then, in human AAA transcriptome datasets, the high expression of *CLEC4E*, *IL1B* was confirmed and a logistic regression model based on the two gene expressions was built, with an AUC of 0.9 in the train set and 0.79 in the validated set. Additionally, in our cohort, we confirmed the increased serum protein levels of IL-1β and CLEC4E in AAA patients as well as the increased expression of these two genes in AAA aorta samples.

**Conclusion:**

This study identified monocytes/macrophages as the main immune cells infiltrated into the early-stage AAA and constructed a logistic regression model based on monocytes/macrophages related gene set. This study could aid in the early diagnostic of AAA.

## Introduction

Abdominal aortic aneurysm (AAA) was defined as having diameters 1.5 times greater than normal (or which measure > 3 cm), which is a life-threatening aortic disease characterized by permanent, localized dilations of the abdominal aorta. AAA is an important cause of morbidity and mortality in developed countries (1). AAA rupture is a leading cause of death, an AAA might be asymptomatic until it ruptures (2). Early detection of AAA is therefore critical. Currently, ultrasonography is the most effective method of choice for early diagnosis of AAA (3). Given the cost-effectiveness of screening, the development of novel biomarkers for the detection of early AAA appears to be a viable future undertaking (4). In this context, understanding the molecular mechanism of early AAA pathogenesis is crucial.

Generally, apoptosis of smooth muscle cells, degradation of the extracellular matrix, infiltration of inflammatory cells, and increase of oxidative stress were considered to be central parts of AAA pathogenesis (5). With the deepening of research, growing evidence emerged indicating the invasion of diverse immune cells, such as macrophages, CD4^+^ T cells, NK cells, and others, played a significant role in the development of AAA (6). Furthermore, the infiltration of immune cells into the aortic wall was discovered to occur early in the development of AAA (7). Understanding the process of immune infiltration is therefore critical for devising AAA medication therapy and developing early diagnostic methods.

Several animal models have been established in recent decades to examine the mechanisms involved in the formation and progression of AAA, and each animal model has its own benefits in reflecting distinct aspects of AAA (8). Angiotensin II infusion model, elastase perfusion model, and CaCl_2_ perivascularly application model were the three most widely used AAA animal models as they were stable, easily accessible, and can reflect representative features of AAA pathogenesis, including early-stage inflammatory response and apoptosis of smooth muscle cells (9). Experimental animal models are of great significance for understanding the pathogenesis of early AAA.

With the advancement of high throughput sequencing technology, increasing amounts of biological data have been generated, and recently, scRNA-sequence was applied to study the mechanism of AAA progress, providing new insights into the disease’s etiology (10). Based on the large scale of data and various bioinformatic methodologies, several studies investigated the differential gene expression pattern and immune infiltration pattern of AAA. However, few studies focus on the early stage of AAA growth. In the present study, we download several scRNA-seq datasets and microarray datasets from the early stage of experimental AAA to screen for potential biomarkers by various bioinformatics analysis methods.

## Materials and methods

### Data collection and processing

In our study, we downloaded scRNA-seq dataset GSE152583, GSE164678, GSE166676 and mRNA expression dataset GSE51227, GSE109639, GSE17901, GSE57691, GSE47472 from Gene Expression Omnibus (GEO)^1^ database. Table 1 showed the details of the datasets used.

**TABLE 1:** Details of the datasets used in this study.

Dataset	Type	Platform	Sample species	Samples included and stage
GSE152583 (35)	scRNA-seq	Illumina HiSeq 4000	Mouse [Elastase-induced AAA model, peri-adventitial elastase incubation)]	Control (*N* = 1, 5 pooled aortas); AAA (*N* = 1, 5 pooled aortas, days 7 post induced)
GSE164678 (36)	scRNA-seq	Illumina NovaSeq 6000	Mouse (CaCl_2_-induced AAA model)	Control (*N* = 1, 4 pooled aortas); AAA (*N* = 1, 4 pooled aortas, days 4 post induced)
GSE51227 (37)	Microarray	Agilent-028005 SurePrint G3 Mouse GE 8 × 60 K Microarray	Mouse (Elastase-induced AAA model, intraluminal perfusion)	Control (*N* = 5); AAA (*N* = 5, days 7 post induced)
GSE109639 (38)	Microarray		Mouse (CaCl_2_-induced AAA model)	Control (*N* = 3); AAA (*N* = 3, days 7 post induced)
GSE17901 (39)	Microarray	Agilent-014868 Whole Mouse Genome Microarray 4 × 44K G4122F	Mouse (AngII-induced AAA model)	Control (*N* = 6); AAA (*N* = 7, days 7 post induced)
GSE166676 (10)	scRNA-seq	Illumina NovaSeq 6000	Human	Control (*N* = 2); AAA (*N* = 4)
GSE57691 (40)	Microarray	Illumina HumanHT-12 V4.0 expression bead chip	Human	Control (*N* = 10); AAA (*N* = 49)
GSE47472 (41)	Microarray		Human (AAA neck)	Control(*N* = 8); AAA (*N* = 14)

For datasets GSE152583 and GSE164678, R package “Seurat” v4.0 was used for quality control, normalization, CCA integration, and TSNE dimensional reduction. Cells were filtered out by nFeature_RNA < 200, nFeature_RNA > 4,000, nCount_RNA > 25,000, and percent. mt > 10. After dimensional reduction, marker genes for different cell types were used for cluster identification, and the marker genes used were consistent with the original publications (Supplementary Figures 1A–D). For GSE152583, data from days 0 and 7 samples were subset for further analysis. For dataset GSE166676, cells were filtered out by nFeature_RNA < 200, nFeature_RNA > 2,500, and percent.mt > 25. After dimensional reduction, we just examined the expression pattern of four genes using Featureplot function, and no further step was carried out.

For datasets GSE51227, GSE109639, GSE17091, GSE57691, and GSE47472, R package “Limma” was used for data normalization. Samples from AOD patients in GSE57691 were excluded as those samples were not relevant to the purpose of our study. Boxplots were generated to confirm the normalization effect by R package “ggplot2” (Supplementary Figures 1E–G).

### Immune cell infiltration analysis

Two methods were used to evaluated the infiltration of immune cells. One was Microenvironment Cell Population counter (mMCP-counter), a method developed recently to quantify immune cell populations for the mouse, was employed to evaluate infiltration of immune cells for the mouse aorta samples by using R package “mMCPcounter” (11). The other method used was *CIBERSORT*, with a reference gene set came from ImmuCC (12, 13). Then the results were visualized by a box plot generated by R package “ggplot2.”

### The Weighted gene coexpression network analysis

R package “WGCNA” was used for the Weighted Gene coexpression Network Analysis of monocytes/macrophages populations from datasets GSE152583 and GSE164678. Firstly, monocytes/macrophages populations gene expression matrices were subset, and then we constructed pseudo cells by combining cells in the same sample and clusters. Ten cells were combined as one pseudo cell. After that, high variable genes were selected for further analysis.

### Functional enrichment analysis and protein-protein interaction network construction

The R package “clusterProfiler” (version 4.0.2) was adopted for the Kyoto Encyclopedia of Genes and Genomes (KEGG) pathway enrichment analysis and Gene Ontology (GO) functional annotation to explore the biological functions of genes in the modules that were associated with the disease. By setting up cor.geneModuleMembership > 0.8 and cor.geneTraitSignificance > 0.4 for dataset GSE152583, cor.geneModuleMembership > 0.8 and cor.geneTraitSignificance > 0.3 for dataset GSE164678, hub gene in the modules were selected. Then the PPI network of hub genes was constructed by the STRING database^2^ with a confidence score of 0.4, and the disconnected nodes in the network were hidden.

### Differential gene expression analysis

According to previous comparative analysis, Limma-voom was an ideal method for DEG analysis of scRNA-seq data (14). Thus, the Differentially Expressed Genes (DEGs) of the monocytes/macrophages population in GSE152583 and GSE164678 datasets were explored by the Limma-voom method using R package “edgeR.” log_2_ | FC| ≥ 1 and *P* < 0.05 were set as cut-offs for GSE152583, while FC ≥ 1.3, FC ≤ 0.7 and *P* < 0.05 were set as cut-offs for GSE164678, and the results were visualized by volcano map using R package “ggscatter.”

### Logistic regression model

Multivariate logistic regression analysis was performed using the glm function in R package “stats.” AAA samples and control samples were used as categorical responsive values, and gene expression values were used as continuous predictive variables. Visualization of logistic regression analysis by dynamic nomogram was constructed through R package “DynNom.” Hosmer-Lemeshow goodness-of-fit test was used for calibration examination. Receiver operating characteristic (ROC) curve analysis was generated to evaluate the model to distinguish AAA and normal aorta samples by R package “pROC.”

### Sample collection

For the protein-specific enzyme-linked immunosorbent assay (ELISA), a total of 38 patients diagnosed as AAA and 18 age and gender matched healthy controls were enrolled in the study from the First Hospital of China Medical University. The diagnosis of all patients was confirmed by computed tomography angiography (CTA), The exclusion criteria included subjects with chronic aortic dissection, congenital heart disease, severe vascular stenosis, autoimmune diseases, infectious diseases, malignant tumors, hematological system diseases, previous aortic surgery or received non-steroidal anti-inflammatory drugs or steroids. Approximately 5 mL fasting blood sample was collected from each participant using standardized sterile tubes. All samples were centrifuged immediately at 3,000 r/min for 10 min at 4°C, and the serum was separated, and stored at −80°C until analysis.

For the mRNA expression detection, 10 patients diagnosed as AAA and 10 age and gender matched healthy controls were enrolled in the study. Fresh infrarenal AAA wall tissue samples were collected from patients undergoing open elective aneurysmectomy, and control infrarenal aortas were obtained from organ donors. All the aortic tissues were put into liquid nitrogen in 30 min after collection and stored at −80°C.

Written and informed consent to participate in this study was obtained from all subjects. The baseline characteristic data of the subject involved are presented in Supplementary Table 1. Ethical approval was obtained from the ethical committee of the hospital. The patients/participants provided their written informed consent to participate in this study.

### Real-time quantitative polymerase chain reaction and enzyme linked immunosorbent assay

Aortic specimens were ground in liquid nitrogen, and *RNAiso Plus* reagent (Takara 9109, Shiga, Japan) was used to extract total RNAs, and the concentration and purity of total RNA were detected by a nanometer photometer (IMPLEN). After that, reverse transcription was performed using PrimeScript RT reagent Kit with gDNA Eraser (TaKaRa RR047A, Shiga, Japan). Real-time quantitative polymerase chain reaction (RT-qPCR) was conducted using TB Green^®^ Premix Ex Taq (TaKaRa RR420A, Shiga, Japan) on an ABI Q3 7500 Real-Time PCR System (ABI). *ACTB* was used as internal controls, and the relative expression level of the target gene was calculated as 2−ΔCt, where −ΔCt = (Ct, target gene − Ct, *ACTB*). Statistical analysis was conducted with GraphPad Primer 8.0 (GraphPad Software Inc., GraphPad Prism 8.0.1.2). Primer sequences used in the study were listed in Supplementary Table 3.

IL-1β and CLEC4E concentrations in the serum were measured using a commercial ELISA kit according to the manufacturer’s instructions (#KET6013, EliKine Human IL-1β ELISA Kit; Abbkine Scientific Co., Ltd., Wuhan, China; # EK3805, Human C-type lectin domain family 4 member E ELISA Kit; Sabbiotech, College Park, MD, United States).

### Statistical analyses

All statistical analyses were completed in the R language (Version 4.0.2). The continuous variables were presented as means ± standard deviations. The Shapiro-Wilk normality test was used to test whether the continuous variables conformed to a normal distribution. The Mann–Whitney *U*-test was used for pairwise comparison of data that did not followed a normal distribution, while the Student’s *t*-test was used to evaluate normally distributed data. Correlation analyses were performed by the Spearman test and visualized by R package “corrplot.” *P* < 0.05 was considered statistically significant.

## Results

### Monocytes/macrophages are the main infiltrating immune cells in the early stages of experimental abdominal aortic aneurysms

**FIGURE 1:**
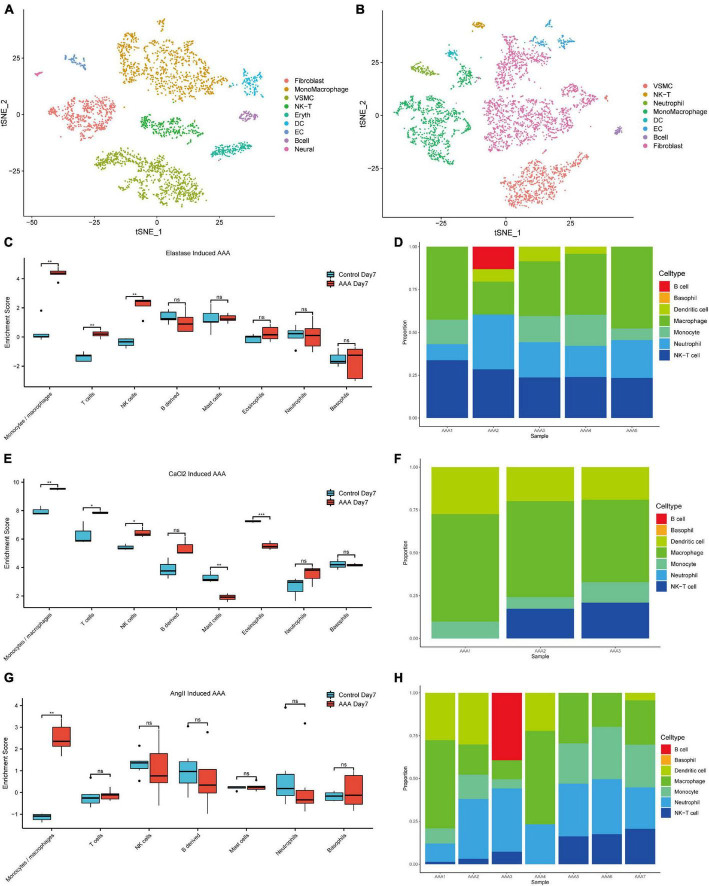
Immune cell infiltration analysis of the early-stage experimental AAA models. The t-SNE plot for peri-adventitial elastase incubation induced AAA scRNA-seq dataset (GSE152583) which contains elastase-induced AAA samples on day 7 and control aortas **(A)**. The t-SNE plot for CaCl_2_ induced AAA scRNA-seq dataset (GSE164678) which contains CaCl_2_-induced AAA samples on day 4 and control aortas **(B)**. Boxplot of the mMCP-counter enrichment score of microarray dataset **(C)** GSE51227 which contains AAA samples induced by intraluminal elastase perfusion on day 7 and control aortas **(E)** GSE109639 which contains CaCl_2_-induced AAA samples on day 7 and control aortas **(G)** GSE17901 which contains AAA samples from AngII treated ApoE^–/–^ mice on day 7 and control aortas. Bar graph of the CIBERSORT enrichment ratio of microarray dataset GSE51227 **(D)**, GSE109639 **(F)**, GSE17901 **(H)** (*P < 0.05, **P < 0.01, ***P < 0.001, ns, not significant).

After data-processing, we clustered all the cells into 17 cell clusters from the peri-adventitial elastase incubation induced AAA dataset (GSE152583), then we identified these clusters by marker genes and classified them into 10 different cell types, which were monocytes/macrophages (4 clusters), smooth muscle cells (4 clusters), fibroblasts (2 clusters), NK-T cells (2 clusters), endothelial cells (1 cluster), dendritic cells (1 cluster), B cells (1 cluster), erythrocytes (1 cluster) and neural cells (1 cluster). Similarly, from the CaCl_2_ induced AAA dataset (GSE164678), we got 15 cell clusters and classified them as monocytes/macrophages (4 clusters), fibroblasts (4 clusters), smooth muscle cells (2 clusters), B cells (1 cluster), endothelial cells (1 cluster), dendritic cells (1 cluster), NK-T cells (1 cluster), and neutrophils (1 cluster) (Figures 1A,B). Despite the different methods of inducing the AAA model, in both datasets, the TSNE plot showed that monocytes/macrophages accounted for the majority of immune cells infiltrating the aorta.

At the same time, deconvolution algorithms were used to evaluate the immune infiltration landscape from three bulk RNA-seq datasets that contain early-stage AAA samples (day 7) created by three different methods. Microenvironment Cell Population counter (mMCP-counter) immune infiltration analysis was used to analyze the differences of the immune cell infiltration levels between different early-stage AAA and control samples. As shown in Figures 1C,E,G, monocytes/macrophages cells got much higher enrichment scores in the early stage AAA samples than that in the control aorta for the three datasets. Moreover, *CIBERSORT* was used to estimate the immune cell composition in the sample, among the various immune cell types, monocytes and macrophages got the most enrichment ratios in the early stage AAA samples (Figures 1D,F,H). All the results above highlighted the vital role of monocytes/macrophages in the early stage of AAA.

### Weighted gene coexpression network analysis

**FIGURE 2:**
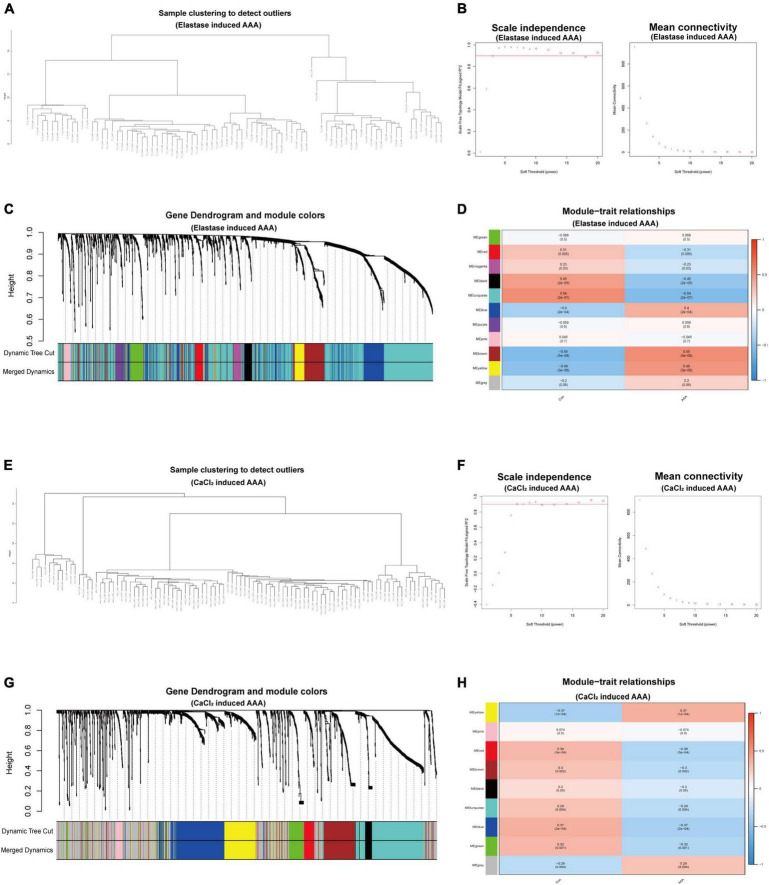
WGCNA for monocytes/macrophages populations of scRNA-seq datasets. WGCNA for monocytes/macrophages populations from peri-adventitial elastase incubation induced AAA dataset (GSE152583) **(A–D)** and CaCl_2_ induced AAA dataset (GSE164678) **(E–H)**. Sample clustering dendrogram for the pseudocells **(A,E)**. Topology network analysis of the scale-free fit index for various soft-thresholding powers (β) and the mean connectivity for various soft-thresholding powers **(B,F)**. The cluster dendrograms represented the co-expression modules **(C,G)**. Heatmap exhibited the relationships between gene modules and clinical traits (Con and AAA) by Spearman correlation **(D,H)**.

To further investigate the gene expression patterns of monocytes/macrophages between the early-stage AAA and control aorta, we extracted the expression matrix of monocytes/macrophages populations from the two scRNA-seq datasets and then constructed pseudo cells with highly variant genes to perform WGCNA analysis. After the combination of cells, 98 pseudocells with 1,891 high variable genes were developed from 1,093 monocytes/macrophages cells for the CaCl_2_ induced AAA dataset (GSE164678), while for the peri-adventitial elastase incubation induced AAA dataset (GSE152583), 84 pseudocells with 1,960 high variable genes were generated from 938 monocytes/macrophages cells. Sample clustering dendrogram for the pseudocells generated from the two datasets was shown in Figures 2A,E. After that, step by step process was used to generate a co-expression network. For the elastase induced AAA dataset, β was chosen to be 3 (*R*^2^ = 0.9) to comply with the scale-free network, and β = 6 was used for the CaCl_2_ induced AAA dataset (Figures 2B,F). The genes were grouped into 11 modules in the macrophage gene expression matrix of the elastase induced AAA dataset and 9 modules in the CaCl_2_ induced AAA dataset by setting the Cut-off value to 0.25 and the minModuleSize to 30 Figures 2C,G). The Spearman correlation between the module eigen gene and the traits was calculated in a subsequent phase to investigate module-trait correlations. As shown in Figures 2D,H, the blue, brown, and yellow modules were positively correlated with aneurysms in the elastase induced AAA dataset, whereas only the yellow gene module was positively correlated with AAA development in the CaCl_2_ induced AAA dataset.

### Functional enrichment analysis and protein-protein interaction analysis of gene module

**FIGURE 3:**
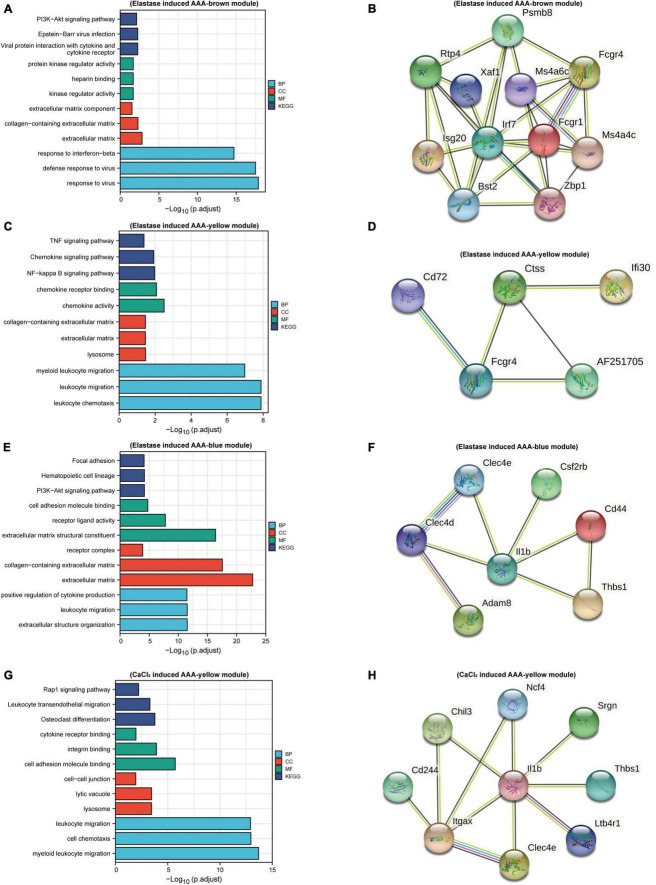
Function enrichment and PPI analysis. Bar plots of GO and KEGG function enrichment results for genes in brown module **(A)**, yellow module **(C)**, and blue module **(E)** of peri-adventitial elastase incubation induced AAA dataset (GSE152583) and genes in blue module **(G)** of CaCl_2_ induced AAA dataset (GSE164678). PPI network for hub genes in brown module **(B)**, yellow module **(D)**, and blue module **(F)** of elastase induced AAA dataset and for hub genes in blue module **(H)** of CaCl_2_ induced AAA dataset.

To understand the function of the modules, GO, and KEGG functional annotation analyses were performed using genes in the modules. For the brown module in the elastase induced AAA dataset (GSE152583), GO analysis revealed that genes within the module are associated with the following BPs including “response to interferon-beta,” “defense response to virus,” and “response to Virus”; the following CCs including “extracellular Matrix,” “collagen-containing extracellular matrix” and “extracellular matrix component”; the following MFs including “protein kinase regulator activity,” “heparin binding” and “kinase regulator activity.” While, KEGG enrichment analysis revealed genes in the module were associated with “PI3K-Akt signaling pathway,” “Epstein-Barr virus infection” and “Viral protein interaction with cytokine and cytokine receptor” (Figure 3A). In addition, there are 13 hub genes identified in the brown module, including *Xaf1*, *Bst2*, *Irf7*, *Ms4a6c*, *Mnda*, *Ms4a4c*, *Fcgr1*, *Phf11b*, *Zbp1*, *Psmb8*, *Isg20*, *Rtp4*, *Fcgr4*, and the PPI network of those genes was shown in Figure 3B.

As for the yellow module in the elastase induced AAA dataset, genes in this module were involved in “myeloid leukocyte migration,” “leukocyte migration” and “leukocyte chemotaxis” for BPs; “lysosome,” “extracellular matrix” and “collagen-containing extracellular matrix” for CCs; “chemokine receptor binding,” “chemokine activity” for MFs. What’s more, KEGG enrichment analysis revealed genes in the module were associated with “NF-Kappa B Signaling Pathway,” “Chemokine Signaling Pathway” and “TNF signaling pathway” (Figure 3C). *Cxcl16*, *Ifi30*, *AF251705*, *Ctss*, *Cd72*, *Fcgr4* were identified as hub genes for the yellow module. Figure 3D showed the PPI network of hub genes in the yellow module.

In terms of genes within the blue module, “positive regulation of cytokine production,” “leukocyte migration,” “extracellular structure organization” were the most enriched pathways for BPs; “extracellular matrix structural constituent,” “receptor ligand activity,” and “cell adhesion molecule binding” were the most enriched for CCs; while “extracellular matrix,” “collagen-containing extracellular matrix” and “receptor complex” were the most enriched pathways for MFs. Meanwhile, “PI3K-Akt signaling pathway,” “Focal adhesion” and “Cytokine-cytokine receptor interaction” were the most enriched pathways for KEGG analysis (Figure 3E). Hub genes in this module included *Thbs1*, *Csf2rb*, *Il1b*, *Cd44*, *Clec4d*, *Adam8*, *and Clec4e*. PPI network of hub genes in this module is shown in Figure 3F.

As mentioned above, for the CaCl_2_ induced AAA dataset (GSE164678), only the yellow module was positively associated with AAA development. GO enrichment analysis revealed that genes in this module were associated with “cell Chemotaxis,” “myeloid leukocyte migration,” and “leukocyte migration” for BPs; “lysosome,” “lytic vacuole,” and “cell-cell junction” for CCs, “cell adhesion molecule binding,” “integrin binding” and “cytokine receptor binding” for MFs. KEGG enrichment analysis revealed “Rap1 signaling pathway,” “Osteoclast differentiation” and “Leukocyte transendothelial migration” pathways were related to genes in this module (Figure 3G). 12 hub genes meeting our criterion were selected, including *Cd244*, *Gngt2*, *Thbs1*, *Chil3*, *Srgn*, *Itgax*, *Ltb4r1*, *Spint1*, *Il1b*, *Clec4e*, *Ncf4*, *Trem3*, and PPI network of hub genes in the gene module was shown in Figure 3H.

### Identification of key monocytes/macrophages related gene set

**FIGURE 4:**
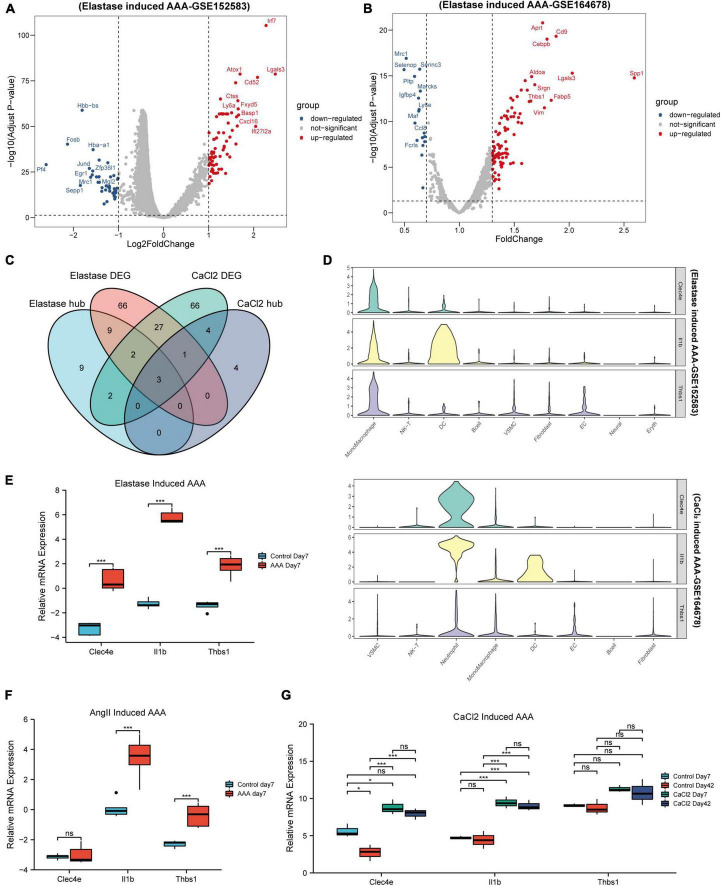
Identification of key genes. Volcano map of differential genes for monocytes/macrophages populations between control and AAA model group in peri-adventitial elastase incubation induced AAA dataset (GSE152583) **(A)** and CaCl_2_ induced AAA dataset (GSE164678) **(B)**. Veen diagrams of DEGs and hub genes for monocytes/macrophages populations between elastase induced AAA dataset and CaCl_2_ induced AAA dataset **(C)**. Violin plots of *Clec4e*, *Il1b*, and *Thbs1* expression in different cell types from peri-adventitial elastase incubation induced AAA dataset and CaCl_2_ induced AAA dataset **(D)**. Boxplot showing the relative expression of *Clec4e*, *Il1b*, and *Thbs1* in the early stage AAA and control aortas for intraluminal elastase perfusion induced AAA microarray dataset (GSE51227) **(E)** and AngII induced AAA dataset (GSE17091) **(F)**. Boxplot showing the relative expression of *Clec4e*, *Il1b*, and *Thbs1* in AAA and control aortas of day 7 and 42 for CaCl_2_ induced AAA microarray dataset (GSE109639) **(G)**. *P < 0.05, ****P < 0.0001.

As we can observe, the gene expression patterns of monocytes/macrophages in AAA models built using various approaches varied significantly. Next, we wanted to see if, despite diverse AAA models, monocytes/macrophages share changed genes throughout the early stages of AAA onset. So, firstly, differential gene expression analysis between the monocytes/macrophages populations of the control aorta and AAA was performed in the two datasets. As shown in Figures 4A,B, 67 up-regulated DEGs and 41 downregulated DEGs were identified in elastase induced AAA dataset (GSE152583), while 88 up-regulated DEGs and 17 downregulated DEGs were identified in CaCl_2_ induced AAA dataset (GSE164678) (Figures 4A,B and Supplementary Table 2). Then we took the intersection of the hub genes identified above and the DEGs in the two datasets. Finally, three genes, *Thbs1*, *Il1b*, and *Clec4e*, were chosen (Figure 4C). After that, we looked at how the three genes were expressed in distinct cell groups. For the peri-adventitial elastase incubation induced AAA dataset, these three genes were mostly expressed in monocytes/macrophages, while for the CaCl_2_ induced AAA dataset, they were mostly expressed in neutrophils and monocytes/macrophages (Figure 4D). The elevated expression of the three genes was then validated in other datasets (Figures 4E–G). Furthermore, it is worth mentioning that the high expression of the three genes persist until day 42 following CaCl_2_ induction (Figure 4G).

### Logistic regression model for prediction of abdominal aortic aneurysm

**FIGURE 5:**
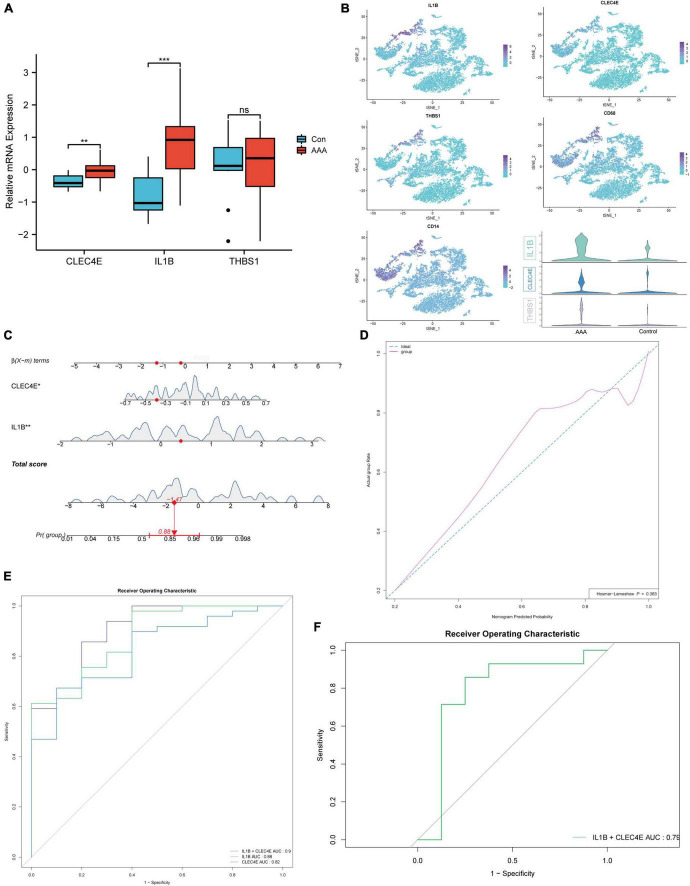
Construction of the logistic regression diagnostic model. Boxplot showing the relative expression of *CLEC4E*, *IL1B*, and *THBS1* in AAA and control aortas for human AAA dataset (GSE57691) **(A)**. *CLEC4E*, *IL1B*, *THBS1*, and *CD68* expression in different cell types of human AAA scRNA-seq dataset (GSE166676) **(B)**. Dynamic nomogram of the two-gene-based model for predicting patients with AAA **(C)**. Dynamic nomogram of the two-gene-based model for predicting patients with AAA **(C)**. The calibration curve of the model **(D)**. ROC curves for the train dataset GSE57691 **(E)** and the validation dataset GSE47472 (human AAA neck) **(F)**. **P < 0.01, ***P < 0.001.

Going a step further, the expression of the three genes in the human AAA dataset was inspected, and all three genes were found to be up-regulated, as shown in Figure 5A. We also discovered that the three genes colocalized with CD68, a common macrophage marker, using the human AAA scRNA-seq dataset (Figure 5B). Next, we wanted to know if these three genes had potential diagnostic value for AAA in humans. So, we built a logistic regression model employing the gene expression in the GSE57691 dataset as continuous predictive variables and sample type (AAA and control aorta) as categorical responsive values. The *P*-values of *IL1B* and *CLEC4E* were < 0.05 in the logistic regression model. Figure 5C shows the dynamic nomogram of the logistic regression model. The calibration curve of the model showed that the predicted probability and the observed probability were generally fitting (Figure 5D), and the *P*-values of the Hosmer-Lemeshow test were > 0.05. The area under the curve (AUC) value was used to assess the discrimination of the models. As a result, the AUC of the combined diagnostic method was 0.9 (Figure 5E). In addition, the GSE47472 dataset, which includes AAA neck samples with less severe lesions, was used to evaluate the effectiveness of this logistic regression model. The AUC value of 0.79 demonstrated the model’s superior ability to distinguish between AAA and control aorta (Figure 5F).

### Validation of key monocytes/macrophages related gene set in human abdominal aortic aneurysm aorta and serum

**FIGURE 6:**
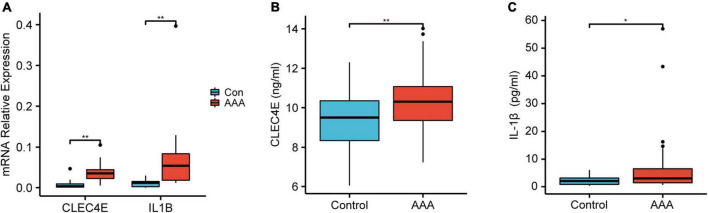
Validation of the gene expression and serum protein level. Boxplot showing the relative expression of *CLEC4E* and *IL1B* in AAA (*N* = 10) and control aortas (*N* = 10) **(A)**. Boxplot showing the protein level of CLEC4E **(B)** and IL-1β **(C)** in AAA (*N* = 30) and control serum (*N* = 10). *P < 0.05, **P < 0.01.

To further validate the high expression of the two genes in human AAA samples, we collected aortic tissues from AAA patients (*n* = 10) and non-AAA aortas from healthy donors (*n* = 10). Patients and the control group were age and sex-matched (Supplementary Table 1). As shown in Figure 6, *IL1B* and *CLEC4E* were highly expressed in the AAA sample. Next, we further detected the proteins expression levels of IL-1β and CLEC4E in the serum of patients. IL-1β expression was detectable in 32 of 38 AAA patients and in 10 of 18 control samples (*P* = 0.044). *IL1B* was increased in AAA patients (7.532 ± 12.529, Mean ± SD, pg/mL) compared with controls (2.234 ± 1.792, Mean ± SD, pg/mL). To our surprise, CLEC4E protein was detectable in the serum of all the participants with a high level of expression. Also, the expression of CLEC4E protein was higher in AAA serum than that in control serum (10.428 ± 1.55 vs. 9.224 ± 1.553, Mean ± SD, ng/mL).

## Discussion

AAA can be asymptomatic in the early stages, but if they reach a late stage, they can rupture and cause abrupt mortality. However, surgical intervention is the only effective method for AAA, and there is no effective medical therapy for patients who do not have surgical indications (15, 16). Understanding the pathology of an AAA in its early stages is critical for early diagnosis, prevention, and therapy.

Previous studies have identified multiple immune cell types in human AAA tissues. To assess the immune infiltration of the early-stage experimental AAA, we employed a deconvolution algorithm based on transcriptome data and cell identification based on single-cell sequencing data. Although some immune cell types, including mast cells, eosinophils, and basophils, had a certain enrichment score through deconvolution evaluation, these cells could not be well identified in the single-cell sequencing data from the same AAA model. This inconsistency may be due to sample variances and single-cell sequencing dropout. In any case, there was good consistency between the main immune cell types that can be identified by single-cell sequencing and the enrichment results of the transcriptome data, including T cells, NK cells, and monocytes/macrophages. Regrettably, no single-cell sequencing data of the early-stage AAA model generated by Ang II is currently available, however, the current study highlighted the importance of monocytes/macrophages in the early stages of AAA. What’s more, several recent studies evaluated the immune infiltration landscape in human AAA samples by *CIBERSORTx*, and the results showed that compared to the control aortas, monocytes and macrophages are the main types of immune cells infiltrated in AAA (17, 18). Hence, the infiltration of monocytes/macrophages may be an event that started in the early stage of AAA and existed through the development of AAA.

WGCNA is one of the most used methods for inferencing gene networks from transcriptomic data. Previous studies have used WGCNA to identify hub genes for AAA based on bulk transcriptomic data (19, 20). But the same gene may have different effects on AAA progression in different cell types. Several studies have shown that WGCNA can be applied to single-cell sequencing data by constructing pseudocells (21, 22). Given the vital role of monocytes/macrophages in AAA immune infiltration, we constructed pseudocells and performed WGCNA based on the monocytes/macrophages expression profile in two different AAA model scRNA-seq datasets. Correlation analysis showed that three gene modules were involved in the early stage AAA induced by elastase. KEGG pathway enrichment analysis showed that the three gene modules were related to virus infection (brown), leukocyte chemotaxis (yellow), and cytokine release (blue), respectively. GO pathway enrichment analysis highlighted the role of “PI3K-Akt signaling pathway,” “NF-Kappa B Signaling Pathway,” “TNF Signaling Pathway” and the importance of extracellular matrix components. Prior studies have noted the vital role of macrophages in ECM degradation, while our study is consistent with this and suggests that this process may be occurring in the early stage of AAA (23). Compared to the elastase-induced AAA model, only one gene module (yellow) was identified as being positively associated with AAA in CaCl_2_ induced AAA, of which pathway enrichment analysis emphasized the role of leukocyte adhesion and Rap1 signaling pathway. DEG analysis of monocytes/macrophages in the CaCl_2_ induced AAA model also showed much less genetic alternations than that induced by elastase, so much so that we had to relax the logFC selection criteria to get the same number of differential expression genes. It is worth mentioning that although CaCl_2_ induced AAA models are widely used, the researchers found that the expansion of AAA was not significant compared to that induced by AngII and elastase, while the aortic calcification was more predominant (24, 25). In line with this, GO pathway enrichment analysis of the yellow gene module in the CaCl_2_ induced AAA model also highlighted the role of osteogenic differentiation, which is a similar physiological process to vascular calcification. Anyway, three genes were identified in both models, including *Thbs1*, *Il1b*, and *Clec4e*. Furthermore, the expression of the three genes in human AAA samples was examined, and a logistic regression diagnostic model was built based on *IL1B* and *CLEC4E*.

*CLEC4E*, also called MINCLE (Macrophage-Inducible C-Type Lectin), is a pattern recognition receptor that belongs to the C-type lectin receptor family. Antigen-presenting cells such as macrophages, neutrophils, DCs, and B cells express MINCLE, which can bind a variety of PAMPs generated from the fungal microbiome, including -mannose, lipidic species, and certain endogenous self-ligands such Sin3A-associated protein 130 (SAP130) (26, 27). In the present study, *CLEC4E* is principally expressed on monocytes/macrophages in elastase-induced AAA model and human AAA samples. As far as we know, there were no studies have investigated the role of MINCLE in the formation of AAA. According to a recent study, Clec4e expressed on macrophages can detect necrotic cells and promote local inflammation, which promotes atherosclerosis (28). Hence, in the early stage of AAA development, MINCLE may be activated by necrotic cells and initiate the early stage inflammatory response.

*IL1B*, which encoded interleukin-1, is a well-known inflammatory gene involved in various diseases. Due to ROS-mediated inflammasome activation, Il-1 was observed to be increased in the early stages of experimental AAA (29, 30). Some researchers suggest that macrophages and vascular smooth muscle cells are the main sources of Il-1β (31). Consistent with the literature, in our study, Il-1β was most expressed on monocytes/macrophages and dendritic cells in elastase-induced AAA model while in CD68^+^ monocytes/macrophages-like cells in human AAA samples. However, though we observed the high expression on monocytes/macrophages in Cacl_2_ induced AAA model, more prominent *IL1B* mRNA expression was observed to express in the neutrophils. Researcher has found that IL-1β expression in neutrophils could contributed to AAA by promoting NETosis during an earlier stage on day 3. Since AAA samples used for sc-RNA sequence by Cacl_2_-induced were taken much earlier (day 4) than that by elastase induced AAA (day 7), we suggest that the difference in *IL1B* expression pattern stems from the different timing of sampling. In AAA patients, elevated plasma and aortic wall Il-1β levels were reported in the previous studies (32, 33). It also has been reported that the Il-1β levels were the same in the AAA patients with small and large aneurysms (maximal diameter > or < 45 mm), highlighting the diagnostic value of Il-1β for AAA in the early stage (34).

There were several limits to our study. First of all, although we used datasets from different AAA models, there was still a certain difference between the pathology of the early-stage experimental AAA and that of human AAA. Secondly, Due to the lack of clinical information in the dataset, risk factors such as gender and smoking status were not considered. Lastly, because it didn’t meet the surgical indications, it was difficult for us to obtain blood and tissue samples from early-stage AAA patients. Thus, we couldn’t evaluate the diagnostic performance of *IL1B* and *CLEC4E* in the early-stage AAA patient cohorts.

## Conclusion

This study downloaded scRNA-seq data and transcriptome data of experimental AAA and human AAA samples from the GEO database. Through multiple bioinformatics analysis methods based on the data, we identified macrophages as the main immune cells infiltrated in the early stage AAA. Moreover, we identified *Clec4e*, *Il1b*, and *Thbs1* as key monocytes/macrophages related genes. After that, a logistic regression diagnostic model was established based on *CLEC4E* and *IL1B*, which can distinguish AAA patients from the control group well.

## Data availability statement

The datasets presented in this study can be found in online repositories. The names of the repository and accession numbers can be found below: https://www.ncbi.nlm.nih.gov/geo/, GSE152583, GSE164678, GSE166676, GSE51227, GSE109639, GSE17091, GSE57691, and GSE47472.

## Ethics statement

The studies involving human participants were reviewed and approved by Ethics Committee at the Chinese Medical University, The First Affiliated Hospital of China Medical University. The patients/participants provided their written informed consent to participate in this study.

## Author contributions

SC and SX designed the study. YL collected the data and materials. SC and YL performed the data analysis. SC wrote the manuscript. YJ and BJ contributed to essential reagents and tools. BJ and SX revised the manuscript. All authors contributed to the article and approved the submitted version.

## Footnotes

1
https://www.ncbi.nlm.nih.gov/geo/

2
http://string-db.org

## Supplementary material

Supplementary Figure 1 Cell marker used for scRNA-seq datasets processing and boxplots of mRNA seq datasets following normalization.

Supplementary Table 1 The baseline characteristic data of the subject involved in our cohort.

Supplementary Table 2 DEGs list of Figure 4.

Supplementary Table 3 Primer sequences used in the work.

## References

[1] Golledge J Muller J Daugherty A Norman P. Abdominal aortic aneurysm: pathogenesis and implications for management. *Arterioscler Thromb Vasc Biol.* (2006) 26:2605–13. 16973970 10.1161/01.ATV.0000245819.32762.cb

[2] Sprynger M Willems M Van Damme H Drieghe B Wautrecht JC Moonen M. Screening program of abdominal aortic aneurysm. *Angiology.* (2019) 70:407–13. 10.1177/0003319718824940 30654619

[3] Schäberle W Leyerer L Schierling W Pfister K. Ultrasound diagnostics of the abdominal aorta: english version. *Gefasschirurgie.* (2015) 20(Suppl. 1):22–7. 10.1007/s00772-014-1411-1 26119947 PMC4479382

[4] Moris D Mantonakis E Avgerinos E Makris M Bakoyiannis C Pikoulis E Novel biomarkers of abdominal aortic aneurysm disease: identifying gaps and dispelling misperceptions. *Biomed Res Int.* (2014) 2014:925840. 10.1155/2014/925840 24967416 PMC4055358

[5] Zhang S-L Du X Chen Y-Q Tan Y-S Liu L. Potential medication treatment according to pathological mechanisms in abdominal aortic aneurysm. *J Cardiovasc Pharmacol.* (2018) 71:46–57. 10.1097/FJC.0000000000000540 28953105

[6] Yuan Z Lu Y Wei J Wu J Yang J Cai Z. Abdominal aortic aneurysm: roles of inflammatory cells. *Front Immunol.* (2020) 11:609161. 10.3389/fimmu.2020.609161 33613530 PMC7886696

[7] Meher AK Spinosa M Davis JP Pope N Laubach VE Su G Novel role of IL (interleukin)-1β in neutrophil extracellular trap formation and abdominal aortic aneurysms. *Arterioscler Thromb Vasc Biol.* (2018) 38:843–53. 10.1161/ATVBAHA.117.309897 29472233 PMC5864548

[8] Golledge J Krishna SM Wang Y. Mouse models for abdominal aortic aneurysm. *Br J Pharmacol.* (2020) 5:792–810. 10.1111/bph.15260 32914434

[9] Patelis N Moris D Schizas D Damaskos C Perrea D Bakoyiannis C Animal models in the research of abdominal aortic aneurysms development. *Physiol Res.* (2017) 66:899–915. 10.33549/physiolres.933579 28937252

[10] Davis FM Tsoi LC Melvin WJ denDekker A Wasikowski R Joshi AD Inhibition of macrophage histone demethylase JMJD3 protects against abdominal aortic aneurysms. *J Exp Med.* (2021) 218:e20201839. 10.1084/jem.20201839 33779682 PMC8008365

[11] Petitprez F Levy S Sun CM Meylan M Linhard C Becht E The murine microenvironment cell population counter method to estimate abundance of tissue-infiltrating immune and stromal cell populations in murine samples using gene expression. *Genome Med.* (2020) 12:86. 10.1186/s13073-020-00783-w 33023656 PMC7541325

[12] Chen Z Huang A Sun J Jiang T Qin FX Wu A. Inference of immune cell composition on the expression profiles of mouse tissue. *Sci Rep.* (2017) 7:40508. 10.1038/srep40508 28084418 PMC5233994

[13] Newman AM Liu CL Green MR Gentles AJ Feng W Xu Y Robust enumeration of cell subsets from tissue expression profiles. *Nat Methods.* (2015) 12:453–7. 10.1038/nmeth.3337 25822800 PMC4739640

[14] Soneson C Robinson MD. Bias, robustness and scalability in single-cell differential expression analysis. *Nat Methods.* (2018) 15:255–61. 10.1038/nmeth.4612 29481549

[15] Chaikof EL Dalman RL Eskandari MK Jackson BM Lee WA Mansour MA The society for vascular surgery practice guidelines on the care of patients with an abdominal aortic aneurysm. *J Vasc Surg.* (2018) 67:2–77.e2. 10.1016/j.jvs.2017.10.044 29268916

[16] Golledge J Moxon JV Singh TP Bown MJ Mani K Wanhainen A. Lack of an effective drug therapy for abdominal aortic aneurysm. *J Intern Med.* (2020) 288:6–22. 10.1111/joim.12958 31278799

[17] Lei C Yang D Chen S Chen W Sun X Wu X Patterns of immune infiltration in stable and raptured abdominal aortic aneurysms: a gene-expression-based retrospective study. *Gene.* (2020) 762:145056. 10.1016/j.gene.2020.145056 32805313

[18] Nie H Qiu J Wen S Zhou W. Combining bioinformatics techniques to study the key immune-related genes in abdominal aortic aneurysm. *Front Genet.* (2020) 11:579215. 10.3389/fgene.2020.579215 33362847 PMC7758434

[19] Kan KJ Guo F Zhu L Pallavi P Sigl M Keese M. Weighted gene co-expression network analysis reveals key genes and potential drugs in abdominal aortic aneurysm. *Biomedicines.* (2021) 9:546. 10.3390/biomedicines9050546 34068179 PMC8152975

[20] Chen S Yang D Liu B Chen Y Ye W Chen M Identification of crucial genes mediating abdominal aortic aneurysm pathogenesis based on gene expression profiling of perivascular adipose tissue by WGCNA. *Ann Transl Med.* (2021) 9:52. 10.21037/atm-20-3758 33553345 PMC7859787

[21] Tosches MA Yamawaki TM Naumann RK Jacobi AA Tushev G Laurent G. Evolution of pallium, hippocampus, and cortical cell types revealed by single-cell transcriptomics in reptiles. *Science.* (2018) 360:881–8. 10.1126/science.aar4237 29724907

[22] Han X Zhou Z Fei L Sun H Wang R Chen Y Construction of a human cell landscape at single-cell level. *Nature.* (2020) 581:303–9. 10.1038/s41586-020-2157-4 32214235

[23] Pyo R Lee JK Shipley JM Curci JA Mao D Ziporin SJ Targeted gene disruption of matrix metalloproteinase-9 (gelatinase b) suppresses development of experimental abdominal aortic aneurysms. *J Clin Invest.* (2000) 105:1641–9. 10.1172/jci8931 10841523 PMC300851

[24] Golledge J. Abdominal aortic aneurysm: update on pathogenesis and medical treatments. *Nat Rev Cardiol.* (2019) 16:225–42. 10.1038/s41569-018-0114-9 30443031

[25] Wang Y Krishna S Golledge J. The calcium chloride-induced rodent model of abdominal aortic aneurysm. *Atherosclerosis.* (2013) 226:29–39. 10.1016/j.atherosclerosis.2012.09.010 23044097

[26] Drouin M Saenz J Chiffoleau EC-. Type lectin-like receptors: head or tail in cell death immunity. *Front Immunol.* (2020) 11:251. 10.3389/fimmu.2020.00251 32133013 PMC7040094

[27] Li TH Liu L Hou YY Shen SN Wang TT C-Type lectin receptor-mediated immune recognition and response of the microbiota in the gut. *Gastroenterol Rep.* (2019) 7:312–21. 10.1093/gastro/goz028 31687150 PMC6821170

[28] Clément M Basatemur G Masters L Baker L Bruneval P Iwawaki T Necrotic cell sensor clec4e promotes a proatherogenic macrophage phenotype through activation of the unfolded protein response. *Circulation.* (2016) 134:1039–51. 10.1161/circulationaha.116.022668 27587433

[29] Usui F Shirasuna K Kimura H Tatsumi K Kawashima A Karasawa T Inflammasome activation by mitochondrial oxidative stress in macrophages leads to the development of angiotensin ii-induced aortic aneurysm. *Arterioscler Thromb Vasc Biol.* (2015) 35:127–36. 10.1161/atvbaha.114.303763 25378412

[30] Sun W Pang Y Liu Z Sun L Liu B Xu M Macrophage inflammasome mediates hyperhomocysteinemia-aggravated abdominal aortic aneurysm. *J Mol Cell Cardiol.* (2015) 81:96–106. 10.1016/j.yjmcc.2015.02.005 25680906

[31] Johnston WF Salmon M Su G Lu G Stone ML Zhao Y genetic and pharmacologic disruption of interleukin-1β signaling inhibits experimental aortic aneurysm formation. *Arterioscler Thromb Vasc Biol.* (2013) 33:294–304. 10.1161/atvbaha.112.300432 23288154 PMC3632435

[32] Wu X Cakmak S Wortmann M Hakimi M Zhang J Böckler D Sex- and disease-specific inflammasome signatures in circulating blood leukocytes of patients with abdominal aortic aneurysm. *Mol Med.* (2016) 22:505–18. 10.2119/molmed.2016.00035 27474483 PMC5072406

[33] Newman KM Jean-Claude J Li H Ramey WG Tilson MD. Cytokines that activate proteolysis are increased in abdominal aortic aneurysms. *Circulation.* (1994) 90:II224–7. 7955258

[34] Juvonen J Surcel HM Satta J Teppo AM Bloigu A Syrjälä H Elevated circulating levels of inflammatory cytokines in patients with abdominal aortic aneurysm. *Arterioscler Thromb Vasc Biol.* (1997) 17:2843–7. 10.1161/01.atv.17.11.2843 9409264

[35] Zhao G Lu H Chang Z Zhao Y Zhu T Chang L Single-cell RNA sequencing reveals the cellular heterogeneity of aneurysmal infrarenal abdominal aorta. *Cardiovasc Res.* (2021) 117:1402–16. 10.1093/cvr/cvaa214 32678909 PMC8064434

[36] Yang H Zhou T Stranz A DeRoo E Liu B. Single-cell RNA sequencing reveals heterogeneity of vascular cells in early stage murine abdominal aortic aneurysm-brief report. *Arterioscler Thromb Vasc Biol.* (2021) 41:1158–66. 10.1161/atvbaha.120.315607 33472403 PMC7904588

[37] Maegdefessel L Spin JM Raaz U Eken SM Toh R Azuma J Mir-24 limits aortic vascular inflammation and murine abdominal aneurysm development. *Nat Commun.* (2014) 5:5214. 10.1038/ncomms6214 25358394 PMC4217126

[38] Furusho A Aoki H Ohno-Urabe S Nishihara M Hirakata S Nishida N Involvement of B cells, immunoglobulins, and syk in the pathogenesis of abdominal aortic aneurysm. *J Am Heart Assoc.* (2018) 7:e007750. 10.1161/jaha.117.007750 29545260 PMC5907549

[39] Spin JM Hsu M Azuma J Tedesco MM Deng A Dyer JS Transcriptional profiling and network analysis of the murine angiotensin II-induced abdominal aortic aneurysm. *Physiol Genomics.* (2011) 43:993–1003. 10.1152/physiolgenomics.00044.2011 21712436 PMC3180735

[40] Biros E Gäbel G Moran CS Schreurs C Lindeman JH Walker PJ Differential gene expression in human abdominal aortic aneurysm and aortic occlusive disease. *Oncotarget.* (2015) 6:12984–96. 10.18632/oncotarget.3848 25944698 PMC4536993

[41] Biros E Moran CS Rush CM Gäbel G Schreurs C Lindeman JH Differential gene expression in the proximal neck of human abdominal aortic aneurysm. *Atherosclerosis.* (2014) 233:211–8. 10.1016/j.atherosclerosis.2013.12.017 24529146

