# Supplementary figures and images for: Identification of key monocytes/macrophages related gene set of the early-stage abdominal aortic aneurysm by integrated bioinformatics analysis and experimental validation
Source: Front Cardiovasc Med. 2022 Sep 14;9:950961. doi: 10.3389/fcvm.2022.950961 (PMC9515382; doi:10.3389/fcvm.2022.950961)

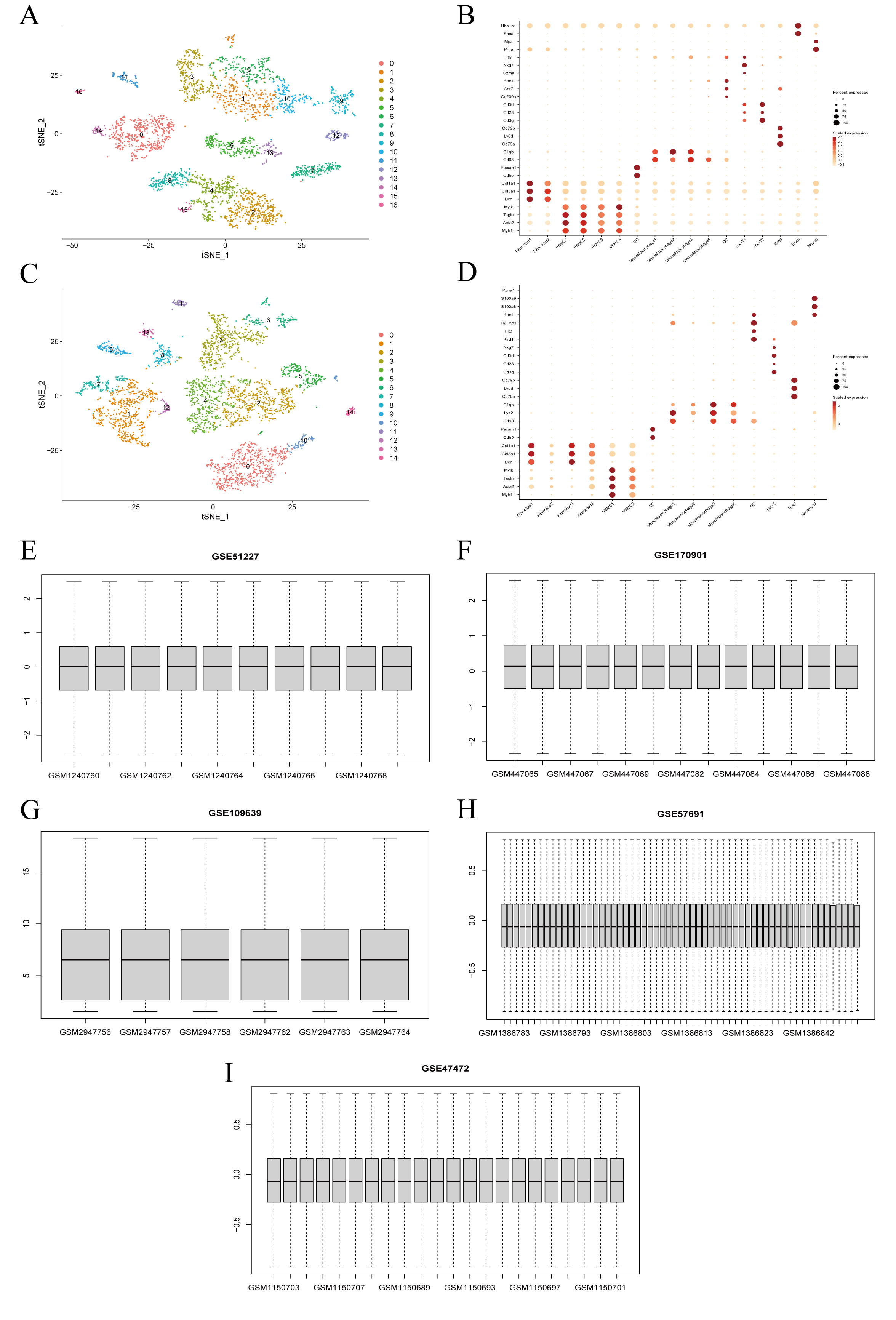

Supplement: Supplementary Figure 1 — Cell marker used for scRNA-seq datasets processing and boxplots of mRNA seq datasets following normalization. [file Image_1.TIF]
